# Influence of Exposure at Different Altitudes on the Executive Function of Plateau Soldiers—Evidence From ERPs and Neural Oscillations

**DOI:** 10.3389/fphys.2021.632058

**Published:** 2021-04-16

**Authors:** Xin Wei, Xiaoli Ni, Shanguang Zhao, Aiping Chi

**Affiliations:** ^1^Institute of Social Psychology, School of Humanities and Social Sciences, Xi'an Jiaotong University, Xi'an, China; ^2^Centre for Sport and Exercise Sciences, University of Malaya, Kuala Lumpur, Malaysia; ^3^School of Sports, Shaanxi Normal University, Xi'an, China

**Keywords:** high altitude, hypobaric hypoxia, SpO_2_, response inhibition, military personnel

## Abstract

This study investigates the changes in soldiers' brain executive function at different altitude environments and their relationship with blood oxygen saturation. Stratified sampling was conducted in different altitude 133 active-duty soldiers who were stationed in Weinan (347 m, *n* = 34), Nyingchi (2,950 m, *n* = 32), Lhasa (3,860 m, *n* = 33), and Nagqu (4,890 m, *n* = 34) for 2 years. The Go/NoGo paradigm with event-related potentials (ERPs) and event-related oscillations (EROs) was used to explore the time and neural oscillation courses of response inhibition. Behavioral results revealed that at the 4,890-m altitude area, the soldiers had the highest false alarm rate, the longest reaction time, and the slowest information transmission rate. The electrophysiological results revealed that NoGo-N2 and N2d decreased with increasing altitude, with significant changes at 3,860 m; the amplitudes of NoGo-P3 and P3d in plateau groups were significantly more negative than the plain and changed significantly at 2,950 m. The results of correlation analysis showed that NoGo-P3 was negatively correlated with altitude (*r* = −0.358, *p* = 0.000), positively correlated with SpO_2_ (*r* = 0.197, *p* = 0.041) and information translation rate (ITR) (*r* = 0.202, *p* = 0.036). P3d was negatively correlated with altitude (*r* = −0.276, *p* = 0.004) and positively correlated with ITR (*r* = 0.228, *p* = 0.018). N2d was negatively correlated with ITR (*r* = 0.204, *p* = 0.034). The power spectrum analysis of NoGo-N2 and NoGo-P3 showed that the power of δ and θ bands at the plateau area was significantly lower than the plain area and showed a significant step-by-step decrease; the α-band power increases significantly only in the area of 4,890 m. The effect of chronic hypoxia exposure at different altitudes of the plateau on the response inhibition of soldiers was manifested: 3,860 m was the altitude at which the brain response inhibition function decreased during the conflict monitoring stage, and 2,950 m was the altitude at which it dropped during the response inhibition stage. In addition, the soldier's brain's executive function was closely related to SpO_2_, and a reduction in SpO_2_ may lead to a decline in response inhibition.

## Introduction

The plateau is in an important strategic military position. Hypobaric and hypoxia are the plateau environment's main characteristics. The brain is the most sensitive organ to hypobaric hypoxia exposure (Heinrich et al., 2019), which can have a significant impact on a variety of cognitive functions, such as long-term exposure that may lead to a decrease in response inhibition (Wang et al., 2014; Davis et al., 2015; Nation et al., 2017).

Neuroimaging studies have proved that high-altitude environment can have significant effects on the human brain. Functional magnetic resonance imaging (fMRI) studies have shown that the activation of the prefrontal cortex (PFC) and anterior cingulate cortex (ACC) is related to response inhibition (Yan et al., 2010). An analysis of the Go/NoGo task found that a significant neural network, including the right superior middle/inferior frontal gyrus, the right lower parietal lobe, and the medial frontal gyrus, was associated with response inhibition (Buchsbaum et al., 2005). Besides, in the study of chronic hypoxia using magnetic resonance imaging (MRI), it was found that a group of subjects born and living at high altitudes had structural changes in the inferior frontal gyrus, middle gyrus, and anterior cingulate cortex (Yan et al., 2011). From altitude on the anterior cingulate cortex and middle frontal gyrus, the response inhibition control-related cortex will be affected, indicating that the plateau's response inhibition control was affected. Response inhibition control is an essential part of cognitive ability and a necessary executive function aspect (Schlaghecken and Eimer, 2006). It focuses on limited cognitive resources on specific target tasks, suppresses some dominant responses, and reduces the impact of habitual behavior or other interference stimuli on target tasks (Palmwood et al., 2017). Then, it improves the efficiency of completing the target task and increases the probability of success. If the individual was affected by the disorder of inhibitory control, there might be behavior out of control and a series of impulsive behaviors. Event-related brain potentials (ERPs) reflect the sum of the brain's neuroelectric responses to specific events with temporal resolution on milliseconds' order (Vinet and Zhedanov, 2011). In Go/NoGo paradigms, the subjects are instructed to respond to repetitive Go stimuli and refrain from responding to infrequent NoGo stimuli (Cheng et al., 2019). There are two main electrophysiological components associated with response inhibition: N2 and P3 (Folstein and Van Petten, 2008; Pires et al., 2014; Fogarty et al., 2018).

Although some of the existing studies focus on the ERPs phase locked in the time domain, little research on the non-phase-locked nerve oscillation reflects the energy change in electroencephalogram (EEG) rhythm when the stimulus was locked. The ongoing information processing can reduce or block the brain waves' amplitude in the α and β rhythms, which is called event-related desynchronization (ERD). Studies have found a positive correlation between ERD intensity and cortical excitation (Daly et al., 2018). ERD activity indicates that the brain is in a state of activation or excitement. The α and β rhythms show increased concussion, called event-related synchronization (ERS) (Pfurtscheller et al., 1997). ERS activity indicates that the brain is in an inactive or resting state. Depending on the characteristics of work and the brain's shape, ERD and ERS can be induced by external or internal events and form a specific brain map distribution. Previous studies have confirmed that ERD and ERS phenomena appear in the process of cognitive information processing (Vázquez-Marrufo et al., 2017).

Therefore, is there no direct evidence that long-term exposure to high-altitude weather affects response inhibition control? If so, is there a regular pattern of the effect of different elevations on response inhibition control? A high-altitude environment can also lead to changes in blood oxygen saturation (SpO_2_) (Brutsaert et al., 2000), and the partial pressure of oxygen decreases with the increase in height. The partial pressure of oxygen in the artery also decreases, leading to a decrease in the artery's SpO_2_ (Bernardi et al., 2017). However, the relationship between SpO_2_ and executive brain function at different elevations remains to be studied.

Therefore, in this study, the Go/NoGo paradigm with event-related potentials (ERPs) and event-related oscillations (EROs) was used to investigate the dynamic changes in brain response inhibition and neuron characteristics oscillation at different altitudes, as well as the relationship between physiological indicators and cognitive components. We hypothesized that the effects of varying altitude plateaus on soldiers' inhibitory control and non-attentive tasks show a progressively decreasing trend change with increasing altitude.

## Subjects and Methods

### Participants

**Figure 1:**
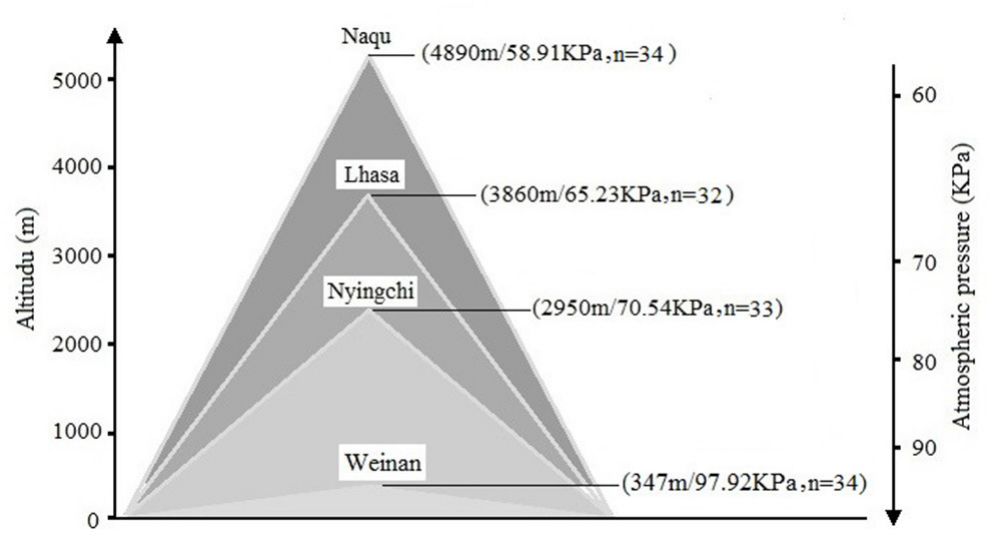
Information on different altitude areas.

Self-compiled general demographic questionnaire was used to register the subject's demographic information, including name, gender, birth date, place of origin, place of residence before enlistment, and education level. A total of 133 soldiers, who were stationed in Weinan (*n* = 34), Nyingchi (*n* = 32), Lhasa (*n* = 33), and Nagqu (*n* = 34) for 2 years, were invited in this study. The mean age of the participants was 22.21 years (*SD* = 0.27). There were no statistical differences for age, body mass index (BMI), and education level among the four groups. More details for the information can be found in Figure 1.

Common inclusion criteria were the following: (1) male, high school education, right-handed (Annett, 1970), normal eye vision or corrected vision, and normal BMI; (2) no head trauma, no physical or mental illness; and (3) no alcohol, drugs, or narcotics within 1 week; and (4) informed consent to participate.

Inclusion criteria for the plateau group (Nyingchi, Lhasa, and Nagqu) were as follows: (1) birthplace was a low-altitude area (<500 m) after stationed in the current altitude area and (2) permanent residence in the current altitude area for 24 months. Exclusion criterion was the experience of going to the plateau before stationing or returning to the plains during stationing. The plain group's inclusion criteria (Weinan) were that the birthplace and stationing place are low altitude (<500 m). Exclusion criterion was an experience of going to the plateau.

The study was conducted following the Declaration of Helsinki, and all procedures were carried out with the adequate understanding and written informed consent of the subjects. The study protocol was approved by the Ethics Committee of the Fourth Military of Medical University.

### General Demographic Information Statistics

#### Measurement of SpO_2_ and HR

The participants' SpO_2_ and heart rate (HR) were measured by an oxygen saturation meter (Kehui Covidien, American). The participants were asked to rest for at least 15 min before measuring. The subjects' right index finger was ensured to be dry and clean without accessories before testing. When calculating, the excessive sunlight affecting the fingers' temperature was avoided, and cold was prevented to ensure the fingers' warmth. When the subjects were breathing calmly, the portable fingertip oxygen saturation meter was used to clip the right hand's index finger. The values of SpO_2_ and HR were read after the reading was stable for at least 10 s.

### Stimuli and Procedures

Participants were seated in a comfortable experimental laboratory and exposed to limited sound and appropriate light. The stimuli were presented on a computer screen ~75 cm away from the participant, with a visual angle of 4° × 4°. Visual stimuli included single and double triangles in the gray background, presented on a computer screen (light degree = 60 cd/m^2^). There were four blocks with 60 Go (double triangles) and 40 NoGo (single triangle) stimuli for each. Before EEG recording, participants performed two practice blocks consisting of 40 Go and NoGo trials to ascertain that their operation was correct. The behavior data such as accuracy rate (AR), false alarm rate (FAR), and reaction time (RT) were collected using E-prime 3.0. Each stimulus was presented for 100 ms, with the mean interstimulus intervals (ISI) being 1,200 ms (randomly between 1,000 and 1,400 ms). During the experiment, participants were instructed to watch the center of the screen, relax, and minimize eye blinks or body movements.

Information transfer rate (ITR) was the standard measurement method of measuring communication and control systems. It refers to the amount of data transmitted per unit of time (Pierce and Epling, 1980). ITR has been widely used in the evaluation of the cognitive attention system. Wolpaw (2002) suggested that ITR be used to evaluate the brain–computer interface's performance. Thus, in this study, ITR was used to assess the effects of cognitive processing rate and hypoxia on soldiers' brains at different altitudes.

ITR is calculated as follows:

$$\begin{array}{l} ITR=\left\{\log_{2}N+P\log_{2}P+\;\left(1-P\right)\log_{2}\frac{1-P}{N-1}\;\right\}/T \end{array}$$

T is the reaction time (RT), N is the total number of stimuli, and P is the accurate rate (AR).

### EEG Acquisition

Brain electric activity was measured from 34 channels using a modified 10–20 system electrode cap (Beijing Yiran Sunny Co. Ltd., www.yiransunny.com.cn). All EEGs were continuously sampled at 1,000 Hz with a left mastoid reference and a forehead ground and referenced offline to an averaged reference derivation by mathematically combining the left and right mastoid. The vertical electrooculogram (EOG) was recorded with electrodes placed above and below the left eye, and the horizontal EOG was recorded with the electrodes placed outboard of both eyes. All electrode impedances were maintained below 5 k*Ω*. EEG and EOG were amplified using a 0.05–100 Hz bandpass and continuously sampled at 1,000 Hz/channel for offline analysis. EEGs contaminated with artifacts due to amplifier clipping, bursts of electromyographic (EMG) activity, or peak-to-peak deflection exceeding ±75 μV were excluded from trials.

### Event-Related Potential Analysis

Raw EEG data preprocessing was performed using EEGLAB software (Version R2013b, San Diego, USA), an open-source toolbox running MATLAB (Version R2013b, MathWorks, United States). The processing process consisted of bilateral mastoid reference and a bandpass filter with a low cut of 0.5 Hz and a high cut at 30 Hz. Eye movement artifacts were corrected using individual independent component analysis (ICA) by removing the corresponding components based on the particular activation curve (Jung et al., 2000; Li et al., 2006). Segments that contained excessive amplitudes (±75) were marked and rejected. The averaged epoch for ERP was 1,000 ms, including a 200-ms prestimuli baseline. The EEGs associated with each stimulus to which subjects correctly responded were overlapped and averaged for each condition.

The peak amplitudes (baseline to peak) of visual N2 and P3 waves were measured in 200–340 ms and 340–430 ms time windows. Furthermore, difference waves were computed by subtracting the average Go-ERP from the average NoGo-ERP for midline electrodes for each condition. As in previous studies (Liu et al., 2009; Zhang et al., 2011), the five electrodes were chosen for statistical analysis: Fz, FCz, Cz, CPz, and Pz. The amplitudes were evaluated by selecting the peak amplitude and computing the mean amplitude over a time window of ±50 ms. The latency onsets were measured with fractional peak latency.

### Event-Related Spectral Perturbation Analysis

The EEG data sampling rate was reduced to 500 Hz, and short-time Fourier transform (STFT) in Matlab was used to analyze EEG data's neural concussion. STFT divides the signal into many identical small-time intervals by window function and uses Fourier transform to explore each time interval to determine the time interval frequency and get a series of variation results of signals in the frequency domain. The baseline of (−200 0) ms corrects the oscillation energy value before stimulation. This study focused on the successful inhibition of N2 and P3 components in NoGo stimulation. STFT was used to calculate the power in five frequency bands: δ (1–4 Hz), θ (4–8 Hz), α (8–14 Hz), β (14–30 Hz), and γ (30–45 Hz).

### Statistical Analysis

All the statistical analyses were performed by SPSS (23.0; SPSS, Inc., Chicago, IL, United States). Kolmogorov–Smirnov test was used to test the normal distribution of each group of data. Comparisons data from the four altitude groups were made using mixed analysis of variance (ANOVA) for normally distributed data. One-way analysis of variance with elevation (Weinan/Nyingchi/Lhasa/Naqu) as a between-subjects factor was conducted for behavior results and physiology indexes. Three-way analysis of variance with elevation (Weinan/Nyingchi/Lhasa/Naqu) as a between-subjects factor and condition (Go, NoGo, NoGo–Go), and electrodeposition (Fz, FCz, Cz, CPz, and Pz) as a within-subjects factor for ERP results. Three-way analysis of variance with elevation (Weinan/Nyingchi/Lhasa/Naqu) as a between-subjects factor and five frequencies bands (δ, θ, α, β, and γ) and electrodeposition (Fz, FCz, Cz, CPz, and Pz) as a within-subjects factor for event-related spectral perturbation (ERSP) results. A *p*-value of 0.05 was considered a significant consequence. The Bonferroni-corrected method was performed for *post-hoc* testing of significant main effects. Effect size in all ANOVA analyses was reported by partial eta-squared (*η*^2^), where 0.05 represents a small effect, 0.10 represents a medium effect, and 0.20 represents a large effect (Faul et al., 2007).

## Results

### Behavior Results and Physiology Measures

The behavioral outcomes can be seen in Table 1. For the Go stimuli, the RT was significantly increased in the Naqu group compared with the plain area (*p* < 0.01). Meanwhile, the false alarm rate and ITR were markedly enhanced in the Naqu group than in the plain group for the NoGo stimuli (*p* < 0.01). The false alarm rate, RT, and ITR in the Naqu group increased than that in the Nyingchi or Lhasa groups (*p* < 0.05 or 0.01) for the NoGo stimuli.

**Table 1:** Behavior results of participants for Go and NoGo stimulations.

**Groups**	**Go**			**NoGo**		
	**Correct rate (%)**	**RT (ms)**	**ITR (bps)**	**False alarm rate (%)**	**RT (ms)**	**ITR (bps)**
Weinan (*n* = 34)	58.33 ± 11.28	153.35 ± 44.31	19.49 ± 2.66	20.58 ± 9.73	143.63 ± 76.29	6.63 ± 5.92
Nyingchi (*n* = 33)	62.20 ± 13.72	174.73 ± 54.57	18.85 ± 3.23	21.97 ± 10.94	141.00 ± 71.78	7.27 ± 6.17
Lhasa (*n* = 32)	62.70 ± 13.23	192.50 ± 75.91	18.87 ± 6.69	24.48 ± 10.88	135.77 ± 65.56	5.07 ± 3.16
Naqu (*n* = 34)	56.27 ± 12.94	204.80 ± 83.73^*^	17.10 ± 10.27	31.75 ± 16.77^**^^##^	185.40 ± 54.82^#^^&^	12.44 ± 11.19^**^^#^^&&^

vs. Weinan:

* *p < 0.05*,

** p < 0.01; vs. Nyingchi:

# *p < 0.05*,

## p < 0.01; vs. Lhasa:

& *p < 0.05*,

&& *p < 0.01*.

**Figure 2:**
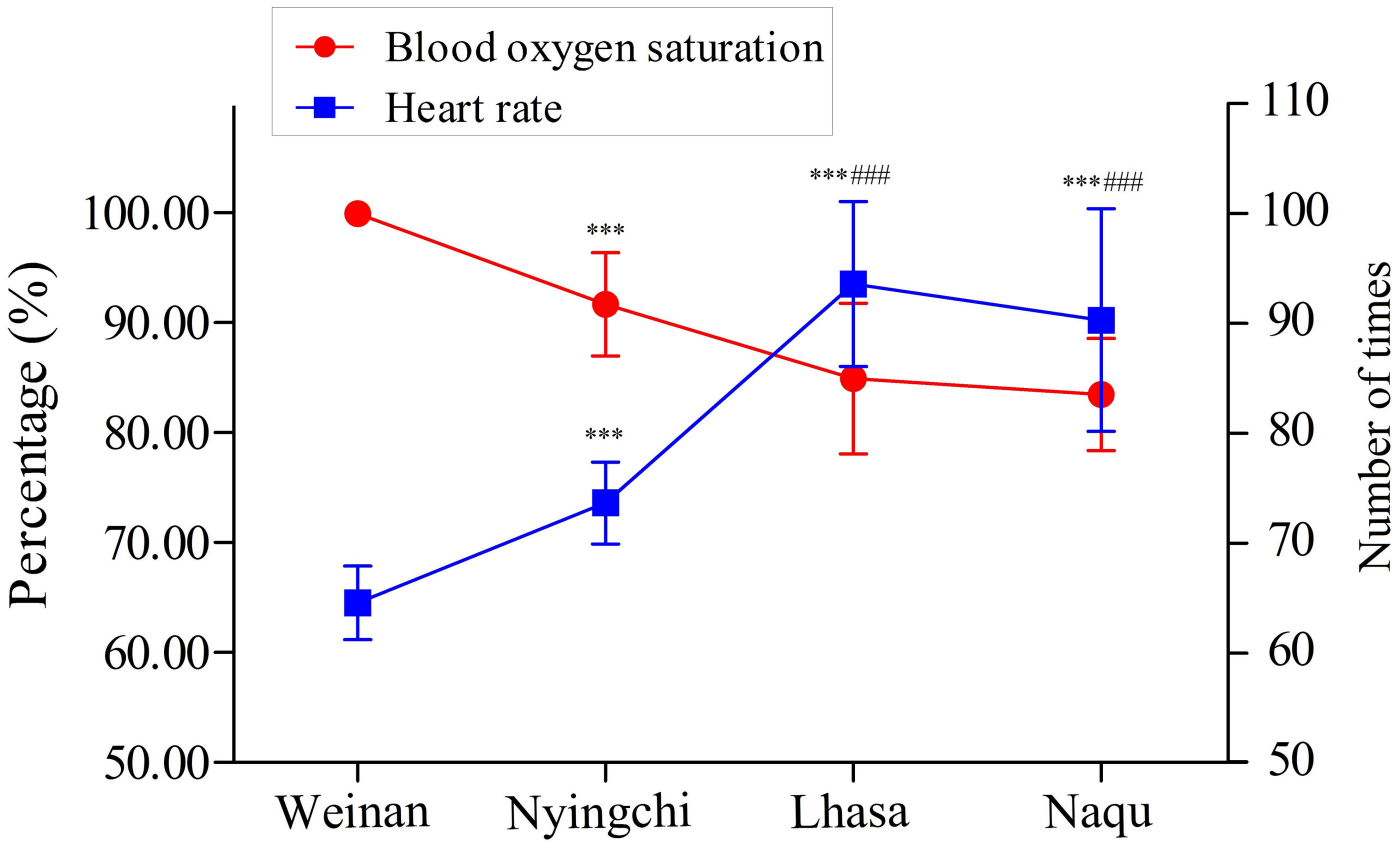
SpO_2_ and HR index of soldiers at different altitudes.

The SpO_2_ and HR of soldiers from four areas are shown in Figure 2. The SpO_2_ percentage was significantly decreased in plateau groups (Nyingchi, Lhasa, and Naqu) compared with the plain group (*p* < 0.001). On the contrary, the soldiers' HR in the three plateau groups markedly increased than that in the plain group (*p* < 0.001). In addition, the SpO_2_ percentage and HR of soldiers in Lhasa and Naqu presented the same changes as Nyingchi (*p* < 0.001). However, no significant difference was found between Lhasa and Naqu.

### ERP Results

#### The Amplitude and Peak Latency of N2

##### N2 Amplitude

A 4(altitude) × 3(trial type) × 5(electrode distribution) mixed analysis of variance was conducted for the peak amplitudes of N200. The main effect of the electrodes was significant [*F*_(4, 1,455)_ = 9.352, *p* < 0.000, *MSE* = 86.49, *η*^2^ = 0.025]. *Post-hoc* analysis revealed that the amplitude of N2 were larger in electrode Fz showing the minimum amplitude (*M* = 2.443 μV, *SE* = 0.273 μV) and electrode CPz showing the maximum amplitude (*M* = 4.600 μV, *SE* = 0.259 μV).

The results revealed interaction effects between altitude and trial type [*F*_(6, 1,455)_ = 15.50, *p* < 0.000, *MSE* = 143.38, η^2^ = 0.05]. Simple main effect analysis found that there was no significant difference under Go condition (*p* = 0.527), but there was difference under NoGo trial [*F*_(24, 1,455)_ = 2.923, *p* = 0.033, *MSE* = 27.036, η^2^ = 0.006] and a significant difference in difference wave N2d [*F*_(24, 1,455)_ = 42.73, *p* < 0.000, *MSE* = 394.95, η^2^ = 0.081]. Further analyses revealed that at NoGo trial, the amplitudes of N2 was positive for Naqu [*M* = 2.671 μV, 95% CL (1.997, 3.064), *p* = 0.019] and Lhasa [*M* = 2.528 μV, 95% CL (2.033, 4.720), *p* = 0.022] regions than that for the plain [*M* = 1.557 μV, 95% CL (1.023, 2.09)]. The amplitudes of N2d for the plain group [*M* = −3.617 μV, 95% CL (−4.239, −3.103)] were negative than that for Lhasa [*M* = −2.235 μV, 95% CL (−2.730, −1.704), *p* = 0.017] and Naqu [*M* = −1.906 μV, 95% CL (−2.44, −1.373), *p* < 0.000].

**Figure 3:**
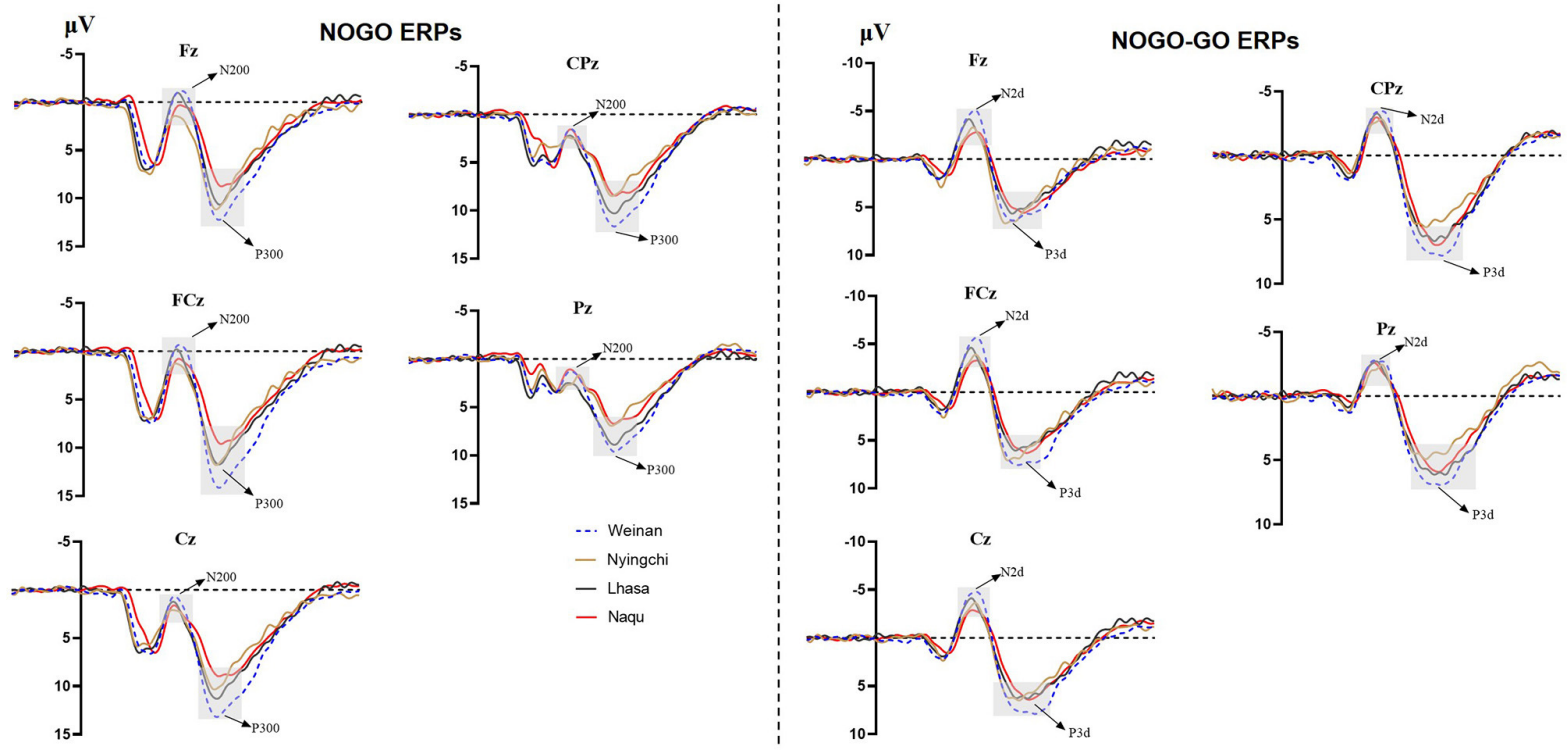
Total average ERP waveform at different altitudes.

There was no interaction effect between altitude and electrodes [*F*_(12, 1,455)_ = 0.618, *p* = 0.829, *MSE* = 5.714, η^2^ = 0.005], no interaction effect between electrodes and trial type [*F*_(8, 1,455)_ = 1.588, *p* = 0.124, *MSE* = 14.683, η^2^ = 0.009], or no triple interaction effects among three variables [*F*_(24, 1,455)_ = 0.276, *p* = 1.00, *MSE* = 2.548, η^2^ = 0.005] (Figure 3).

##### N2 Latency

A 4(altitude) × 3(trial type) × 5(electrode distribution) mixed analysis of variance analysis revealed a significant main effect of electrode on the latencies of N2 [*F*_(24, 1,455)_ = 3.702, *p* = 0.040, η^2^ = 0.049]. The *post-hoc* test suggested that the latency of N2 at Cz electrode was significantly earlier than that of Fz and FCz electrode (265.46 ± 3.48 vs. 270.52 ± 3.43, *p* = 0.034; 265.46 ± 3.48 vs. 269.08 ± 3.25, *p* < 0.001).

The analysis also revealed significant interaction effects between the altitude and trial type [*F*_(4, 1,455)_ = 2.910, *p* = 0.040, η^2^ = 0.108]. Simple effect analysis indicated that there was a significant difference in the NoGo trial [*F*_(12, 1,455)_ = 3.913, *p* = 0.012, η^2^ = 0.140]. The latency of N2 in the Nyingchi group was significantly earlier than that in the plain and the Lhasa groups.

#### The Amplitude and Peak Latency for P3

##### P3 Amplitude

A 4(altitude) × 3(trial type) × 5(electrode distribution) mixed analysis of variance was conducted for the peak amplitudes of P3. The main effect of electrodes was significant [*F*_(4, 1, 037)_ = 7.602, *p* < 0.000, η^2^ = 0.028]. The amplitudes of P3 were positive, with electrode FCz showing the maximum amplitude (*M* = 8.813 μV, *SE* = 0.402 μV) and electrode Pz showing the minimum amplitude (*M* = 6.250, *SE* = 0.405 μV). P3d amplitude were larger, with electrode Fz showing the maximum amplitude (*M* = 6.979 μV, *SE* = 0.337 μV) and electrode Pz showing the minimum amplitude (*M* = 3.409, *SE* = 0.334 μV).

**Figure 4:**
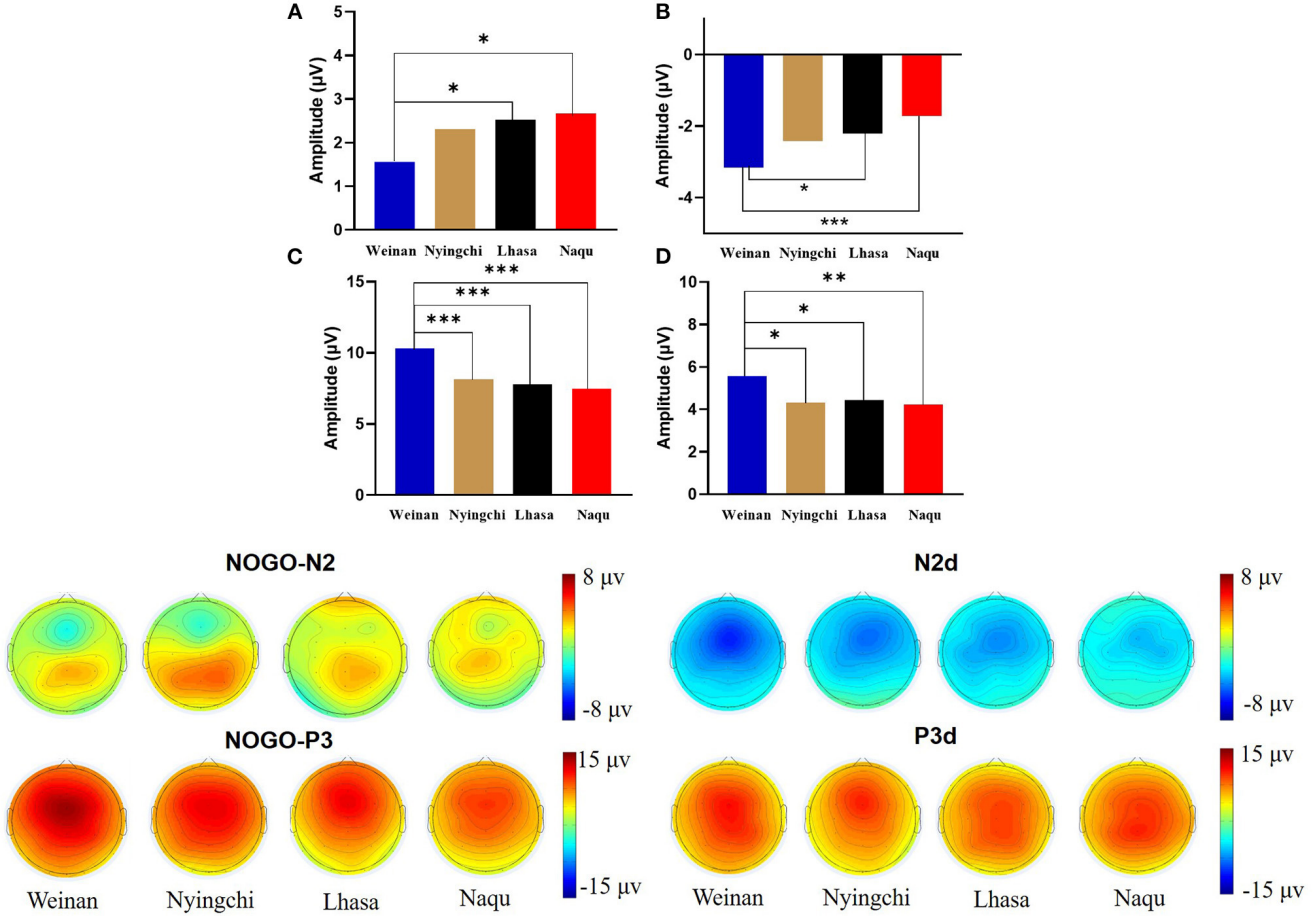
Topographic map of ERP composition at different altitudes.

The results also showed that the interaction effects between altitude and stimulation type were significantly different [*F*_(6, 1,455)_ = 17.361, *p* < 0.000, *MSE* = 280.611, η^2^ = 0.067]. The simple main effect analysis found that there was a significant difference for three conditions at different altitudes [*F*_(3, 1,455)_ = 4.583/74.857/13.88, *p* < 0.01]. A further analysis revealed that at Go trial, the amplitude of P3 in the plain group [*M* = 4.716 μV, 95% CL (4.01, 5.421)] was significantly positive than that in Nyingchi [*M* = 3.202 μV, 95% CL (2.45, 3.954)] and Naqu [*M* = 3.225 μV, 95% CL (2.54, 3.93), *p* = 0.024] groups. In NoGo trial, the amplitude of P3 in the plain group [*M* = 10.295 μV, 95% CL (9.59, 11.00)] was significantly positive than that in Nyingchi [*M* = 7.720 μV, 95% CL (6.968, 8.472)], Lhasa [*M* = 7.799 μV, 95% CL (6.652, 8.37)], and Naqu groups [*M* = 7.464 μV, 95% CL (6.759, 8.17)], *p* < 0.000. The amplitude of P3d were significant positive for the plain [*M* = 6.114 μV, 95% CL (5.408, 6.819)] than for the Nyingchi [*M* = 7.720 μV, 95% CL (6.968, 8.472), *p* = 0.013], Lhasa [*M* = 7.799 μV, 95% CL (6.652, 8.37), *p* = 0.013], and Naqu [*M* = 4.574 μV, 95% CL (3.868, 5.279), *p* = 0.009] (Figure 4).

There was no interaction effect between altitude and electrodes [*F*_(12, 1,455)_ = 0.963, *p* = 0.403, *MSE* = 6.51, η^2^ = 0.003), no interaction effect between trial type and electrodes [*F*_(8, 1,455)_ = 1.435, *p* = 0.177, *MSE* = 23.191, η^2^ = 0.008], or no triple interaction effects among three variables [*F*_(24, 1,455)_ = 0.059, *p* = 1.00, *MSE* = 0.956, η^2^ = 0.005].

##### P3 Peak Latency

A 4(altitude) × 3(trial type) × 5 (electrode distribution) mixed analysis of variance analysis revealed a significant main effect of altitude for the latencies of P3 [*F*_(6, 14, 455)_ = 3.399, *p* = 0.038, η^2^ = 0.124]. The *post-hoc* analysis test suggested that the latency of P3 in the Nyingchi group was significantly earlier than that of the plain, Lhasa, and Naqu groups (355.51 ± 8.15 vs. 386.13 ± 7.52, 378.53 ± 7.52, 387.23 ± 7.71, *p* = 0.007, *p* = 0.042, *p* = 0.006). The analysis also revealed a significant interaction effects between altitude and trial type [*F*_(24, 14, 455)_ = 4.210, *p* = 0.008, η^2^ = 0.149]. Further analysis indicated that at the Go task, the latency of P3 in the Nyingzhi group was significantly earlier than that in the other three groups (*p* < 0.001, *p* = 0.011, *p* = 0.026). At the NoGo task, the latency of P3 in the Nyingzhi group was significantly earlier than that in the other three groups (*p* < 0.001). Other main effects and interaction effects were not significant.

### Correlation Analyses Between Behavioral Data and ERP Components

Pearson correlation analysis revealed that NoGo-P3 and P3d were negatively related to altitudes (*r* = −0.358, *p* < 0.000) and (*r* = −0.276, *p* = 0.004), respectively, and the NoGo-P3 was positively related to SpO_2_ (*r* = 0.197, *p* = 0.041). Correlation analysis also revealed that Go-P3, NoGo-P3, N2d, and P3d were positively related to ITR (*r* = 0.203, *p* = 0.036), (*r* = 0.202, *p* = 0.036), (*r* = 0.204, *p* = 0.034), (*r* = 0.228, *p* = 0.018). The results are shown in Table 2.

**Table 2:** Results of correlation analysis.

	**NoGo-N2**		**NoGo-P3**		**N2d**		**P3d**	
	***r***	***p***	***r***	***p***	***r***	***p***	***r***	***p***
Altitudes	−0.021	0.829	−0.358	**0.000**	−0.062	0.52	−0.276	**0.004**
ITR	−0.022	0.819	0.202	**0.036**	0.204	**0.034**	0.228	**0.018**
SpO_2_	0.020	0.837	0.197	**0.041**	0.015	0.874	0.157	0.104

*Altitudes: Weinan (347 m), Nyingchi (2,895 m), Lhasa (3,890 m), and Naqu (4,860 m)*.

*ITR, information translation rate*.

*The bold indicates a significant correlation*.

### The Results of Frequency Domain (ERO)

#### N2

**Figure 5:**
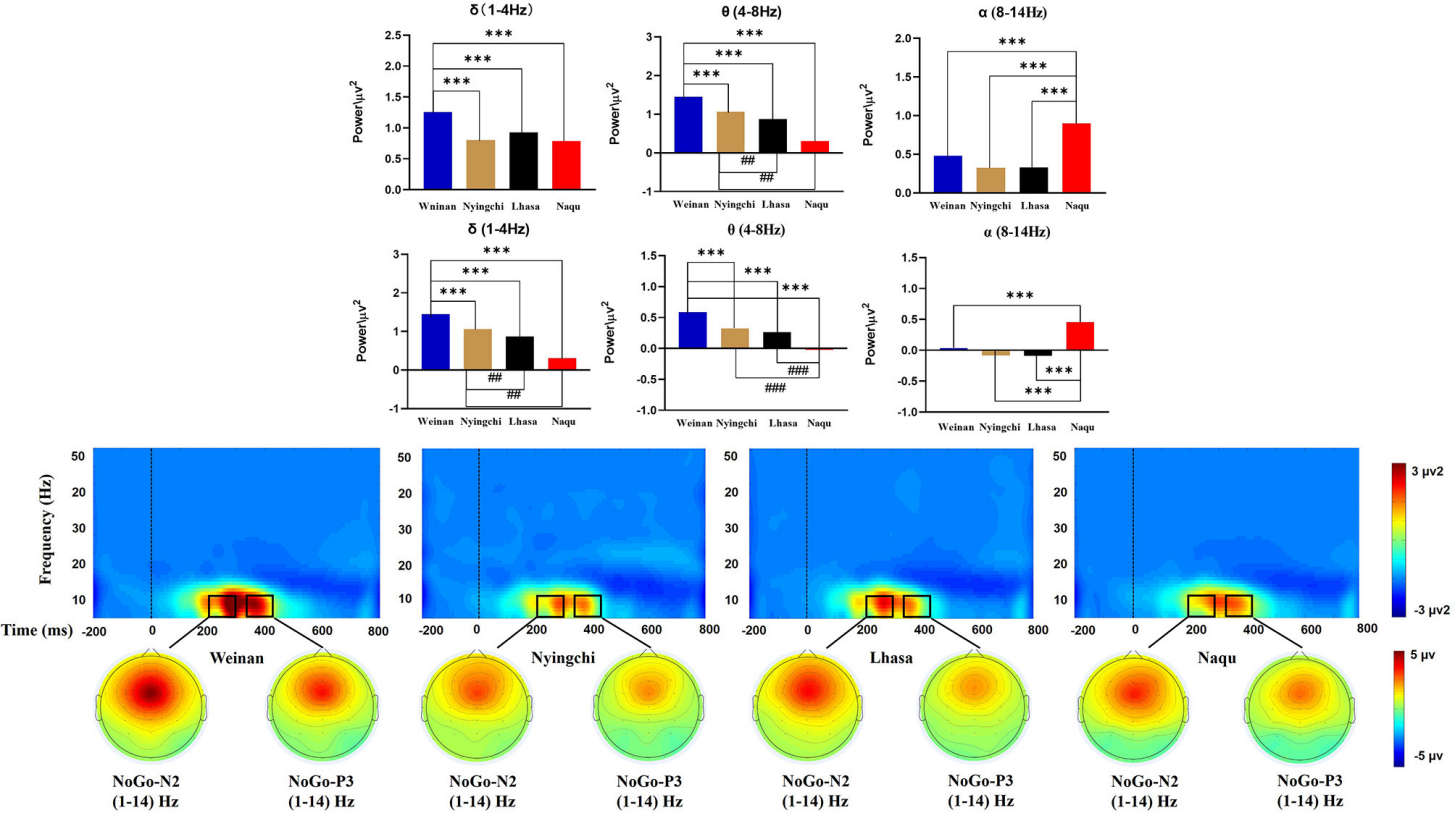
Time-frequency diagram of Nogo stimulation at different altitudes.

A 4(altitude) × 5(frequency band) × 5(electrodes) mixed ANOVA yielded no triple interaction effects [*F*_(48, 2,275)_ = 1.252, *p* = 0.116, *MSE* = 0.449, η^2^ = 0.026]. The results of analysis revealed a significant interaction effect between the altitude and frequency band [*F*_(12, 2,275)_ = 24.599, *p* < 0.000, *MSE* = 8.821, η^2^ = 0.559]. Simple effect analysis found that in the δ, θ, and α bands, there was a significant difference for N2 at different altitudes [*F*_(3, 2,275)_ = 16.359/78.98/24.672, *p* < 0.000]. A further analysis indicated that the power of δ band in the plain group was the highest [*M* = 1.258, 95% CL (1.153, 1.363)], *p* < 0.000], which was significantly larger than that in the Nyingchi [*M* = 0.805, 95% CL (0.693, 0.917)], Lhasa [*M* = 0.926, 95% CL (0.817, 1.036)], and Naqu [*M* = 0.786, 95% CL (0.681, 0.891)] groups. The power of θ band in plain group was the highest [*M* = 1.453, 95% CL (1.347, 1.558), *p* < 0.000], which was significantly larger than that in Nyingchi [*M* = 1.062, 95% CL (0.952, 1.171)], Lhasa [*M* = 0.871, 95% CL (0.759, 0.983)], and Naqu [*M* = 0.307, 95% CL (0.202, 0.412)]. However, the power of α band in the Naqu group was the highest [*M* = 0.9, 95% CL (0.795, 1.01), *p* < 0.000], which was significantly higher than that in the plain group [*M* = 0.479, 95% CL (0.374, 0.584)], Nyingzhi [*M* = 0.329, 95% CL (0.217, 0.441)], and Lhasa [*M* = 0.324, 95% CL (0.215, 0.434)]. The results of analysis also revealed a significant interaction effect between the electrodeposition and frequency band [*F*_(16, 2,275)_ = 14.453, *p* < 0.000, *MSE* = 5.183, η^2^ = 0.092]. Simple effect analysis found that the power of δ, θ, and α bands was the highest at the FCz electrode, decreased gradually at the Fz, Cz, and CPz electrode, and dropped up to the minimum Pz electrode (Figure 5).

#### P3

A 4(altitude) × 5(frequency band) × 5(electrodes) mixed ANOVA yielded no triple interaction effect [*F*_(48, 2,275)_ = 1.073, *p* = 0.341, *MSE* = 0.143, η^2^ = 0.022]. The results of the analysis yielded significant interaction effects between the altitude and frequency band [*F*_(12, 2,275)_ = 29.044, *p* < 0.000, *MSE* = 3.874, η^2^ = 0.133]. Simple main effect analysis found that in the δ, θ, and α bands, there was a significant difference for P3 at different altitudes [*F*_(3, 2,275)_ = 14.741/58.743/59.699, *p* < 0.000]. The power of δ band in the plain group [*M* = 0.657, 95% CL (0.592, 0.721)] was significantly larger than that in the Nyingchi [*M* = 0.38, 95% CL (0.311, 0.448)], and Lhasa [*M* = 0.419, 95% CL (0.353, 0.486)], *p* < 0.000. Naqu [*M* = 0.38, 95% CL (0.311, 0.448)] was significantly larger than Nyingchi and Lhasa, *p* < 0.05. The power of θ band in the plain group was the highest [*M* = 0.589, 95% CL (0.525, 0.653), *p* < 0.000], which was significantly larger than that in Nyingzhi [*M* = 0.261, 95% CL (0.193, 0.33)], Lhasa [*M* = 0.323, 95% CL (0.256, 0.389)], and Naqu [*M* = −0.021, 95% CL (−0.085, 0.043)]. The power of θ band in Naqu was the lowest, which is significantly lower than that in Nyingchi and Lhasa. However, the power of α in Naqu [*M* = 0.453, 95% CL (0.389, 0.517), *p* < 0.000], which was significantly higher than that in Weinan [*M* = 0.035, 95% CL (−0.029, 0.099)], Nyingzhi [*M* = −0.091, 95% CL (−0.159, −0.023)], and Lhasa [*M* = −0.083, 95% CL (−0.149, −0.016)].

## Discussion

### Changes in Response Inhibition Function in Soldiers From Different Areas

In this study, physiological indexes and ERPs were used to explore the dynamic brain changes and neurons' oscillating hypoxia hypobaric environment exposure characteristics to varying altitudes on soldiers' inhibition control ability.

The results showed that the SpO_2_ of soldiers in all three plateau areas was significantly lower than the plain group. The results of HR analysis showed an opposite trend to SpO_2_. Studies have shown that SpO_2_ was negatively correlated with stationing altitude, and HR was positively correlated with stationing altitude (Rivera-Ch et al., 2008; Hilty et al., 2016; Ottestad et al., 2017). These study results are consistent with previous studies' results, and the overall SpO_2_ level and HR of the participants were in the normal range.

The classical Go/NoGo task used in this study presented button stimuli as simple visual stimuli. The RT results showed that both for Go and NoGo stimulation, there was a highly significant increase in the Naqu group compared to the plain group. However, there was no significant difference between the Nyingchi and Lhasa and the plain. The FARs of soldiers in the plateau groups were all higher than that of the plain group, but only the Nagqu and the plain group had significant differences. The analysis of ITR shows that there is no substantial change in the transmission rate from 347 to 3,860 m (Lhasa), but it decreases significantly at the altitude of 4,890 m (Naqu). Previous long-term exposure studies using different cognitive tasks have found that reaction times appear shortened at high altitudes (Richardson et al., 2011). The disappearance of the effects of prolonged exposure on behavior may be due to adaptation supported by compensatory mechanisms. However, the significant increase in the Nagqu area may be due to the decline in executive function caused by prolonged exposure. One study found that the FAR and RT increased in the highland region, indicating that hypoxia exposure led to the decline in executive function (Trompetero et al., 2015). The findings are consistent with the earlier results that the plateau's hypoxic environment has a relatively low effect on simple cognitive tasks. In contrast, the hypoxic environment may impact complex cognitive functions dominated by coordination and accuracy.

The ERPs can reflect information on the neural mechanisms of response inhibition in warriors exposed to high-altitude environments for long periods. In this study, significant response inhibition effects were successfully evoked at four groups, that is, NoGo-N2 and NoGo-P3 components induced by NoGo stimulation were successfully inhibited. Comparing the differences between the three plateau groups and one plain group revealed that the three plateau groups' soldiers showed a significant decrease in amplitude both in the N2 component of conflict monitoring and the P3 component of late processing of information. Studies have indicated that N2 and P3 are considered to function as separable processes in Go/NoGo tasks (Randall and Smith, 2011; Burkhard et al., 2018). The NoGo-N2 component response conflict detection and response inhibition can effectively represent the soldiers' cognitive control degree (Enriquez-Geppert et al., 2010; Burkhard et al., 2018). The NoGo-P3 component amplitude is associated with late processing of response inhibition (Groom and Cragg, 2015). It shows that with the increase in altitude, functional declines in soldiers' response inhibition control become more and more serious, manifested by the decrease in conflict monitoring ability and inhibition control ability in the inhibition processing stage. The amplitude of P3d also decreased significantly in the three plateau groups, especially at the Naqu group. The study showed that the amplitude of P3d was weakened considerably or even missing, suggesting that the function of inhibition control (i.e., response inhibition or movement inhibition) was declined at high altitude. In addition to the response inhibition domain, the P3 component is usually considered the late stage of information processing: the allocated attentional resources (Meinhardt and Pekrun, 2003). It has been shown that high-altitude populations have higher cognitive demands that limit attentional resources for inhibitory control (Hill et al., 2014; Hu et al., 2016). A high-altitude study using an attention task also found lower P3 amplitudes in the high-altitude group than in the low-altitude group (Wang et al., 2014). Chronic high-altitude exposure leads to reduced attentional resource availability (Nieuwenhuis et al., 2005). This study proved that the attention resources of the plateau group decreased compared with the plain control group.

Notably, among the three groups of high-altitude regions, the hypoxic environment in the Nagqu region had the most pronounced effect on the soldiers' inhibitory control function. The primary manifestation was the smallest amplitude of the P3 component of the difference wave, which is considered the neural activity induced by afferent stimuli and reflects the degree of perceptual information processing and an alteration (Godde et al., 2010; Martin et al., 2012; Walsh et al., 2020). This result suggests that a higher altitude leads to a lower degree of inhibitory control activity on information processing due to hypoxia. Studies also show that people's mental and physical working ability in the highland environment is significantly reduced along with the increase in altitude (Tong et al., 2007; Zhu and Fan, 2017). Although the P3 latency is thought to reflect a slowdown in signal processing caused by hypoxia (Ramautar et al., 2006), there was no difference in P3 latency among groups. It may be that the relatively long exposure to high altitude in the subjects of this study may have acclimated to hypoxia. Therefore, the changes in P3 latency at high altitudes found in previous studies may depend on how long individuals live at high altitudes (Mazzeo, 2008). The amplitude of the N2 component, which is a conflict monitor, also tends to weaken, but significant differences are observed in Lhasa at the height of 3,160 m. The results are similar to previous studies' findings that under mild hypoxic conditions, the organism showed increased neural activity, but reduced availability of attentional resources produced neuroexcitatory inhibition, increased impulsivity, and reduced conflict monitoring sensitivity post-conflict processing (Pontifex et al., 2009; Turner et al., 2015; Lefferts et al., 2016; McMorris et al., 2017). The results suggest that cognitive function effects are not easily perceived under mild hypoxic conditions and when complex tasks are not performed. Mental health maintenance should be done in advance, with particular attention to strengthening emotional stability regulation.

### The Variation in δ-, θ-, and α-Band Power in Response Inhibition Function

This study also explored the effects of different altitude plateau on the soldier's response inhibition function near convulsions, focusing on the conflict monitoring and response inhibition phases. Hypoxic exposure can change the neural network's spatial representation and be reflected by EEG's different nervous oscillations (Cvetkovic et al., 2004). The results showed decreased the δ- and θ-band powers of soldiers in three high-altitude areas than those in the plain area. One study showed reduced brain wave activity in the δ and θ bands in a hypoxic environment (more than 30 days) (Zhao et al., 2016). However, the opposite result was found in the simulation module simulating the anoxic environment at high altitude (23 min): the δ and θ bands' activity increased (Papadelis et al., 2007). The reason may be related to the time of hypoxic exposure. This study's subjects are soldiers stationed in various areas for 2 years, indicating that prolonged exposure to a plateau environment can affect brain activity changes. Some studies have explored brainwave activity characteristics in adolescents living at high altitudes and found that δ- and β-frequency activity decreased (Richardson et al., 2011). In this study, there is no difference in the β-frequency activity of soldiers in different regions.

Studies have shown that EEG activity decreases at the beginning of an acute hypoxic exposure and increases with the emergence of α-band activity (Schellart and Reits, 2001; Papadelis et al., 2007). This study shows that the α-band power activity in the conflict monitoring phase of response inhibition function decreases at Nyingchi and Lhasa. There was no significant difference compared with the plain area. However, the α-band power at Naqu was significantly higher than that at the other three groups. This change of power in the specific frequency band of EEG is caused by a decreased synchronization of related nerve oscillations in the cortex (Pfurtscheller and Aranibar, 1979). α-Rhythm synchronization means that this region of the brain is resting or dormant (Pfurtscheller and Aranibar, 1979; Pfurtscheller et al., 1997). α-Rhythmic ERD phenomenon can reveal some pathological states to some extent: some studies have found that Parkinson's patients have α- and β-band ERD phenomenon, showing a hyperactive motor cortex (Heida et al., 2014). Combined with the behavioral results, the above studies further show that soldiers' low information transmission speed in the Naqu was related to weakening of the α-band ERD phenomenon. The study also found that the α-band power in the conflict detection stage of response inhibition was more active than that in the response inhibition stage, indicating that long-term hypobaric hypoxia (HH) exposure caused long-term excitation of the central nervous system, resulting in increased brain loss and gradual decline inactivity. This further reveals that soldiers need to consume more cognitive resources to complete tasks in an anoxic environment.

Several limitations of the present study should be noted. In this study, all the subjects lived at the site for more than 24 months and completed the process of acclimatization at a high altitude. As the experiment was carried out in spring, Nyingchi and Lhasa's oxygen contents are relatively abundant. The sample size involved in the study is relatively small, so the research results may be affected and have some limitations. Second, we did not measure any cerebral oxygen delivery parameters, such as cerebral oxygenation and cerebral blood flow. Future studies should directly measure cerebral oxygenation, cerebral blood flow, and cerebral oxygen delivery and clarify the mechanism of decreased executive function under hypoxia (Ochi et al., 2018).

## Conclusion

In summary, this study found that chronic hypoxia exposure at different altitudes of plateau affects response inhibition, mainly in the conflict monitoring phase and post-processing phase. Further analysis revealed that 3,860 m was the height of decline in the brain's response inhibition function during the conflict monitoring phase, and 2,950 m was the height of decrease during the response inhibition phase. In addition, the soldier's brain executive function was closely related to SpO_2_, and a reduction in SpO_2_ may lead to a decline in response inhibition.

## Data Availability Statement

The original contributions presented in the study are included in the article/supplementary material, further inquiries can be directed to the corresponding author/s.

## Ethics Statement

The study was conducted following the Declaration of Helsinki, and all procedures were carried out with the adequate understanding and written informed consent of the subjects. The study protocol was approved by the Ethics Committee of the Fourth Military of Medical University.

## Author Contributions

XW and SZ were involved in the field for the data collection, troubleshooting, manuscript finalization, organized the data, carried out the analyses, drafted the manuscript, and took the lead in the manuscript finalization and submission. XN and AC were involved in the conceptualization, design, and planning of the study. All authors went through all the versions of the manuscript and approved them.

## Conflict of Interest

The authors declare that the research was conducted in the absence of any commercial or financial relationships that could be construed as a potential conflict of interest.
